# A qualitative case study of child protection issues in the Indian construction industry: investigating the security, health, and interrelated rights of migrant families

**DOI:** 10.1186/1471-2458-13-858

**Published:** 2013-09-17

**Authors:** Theresa S Betancourt, Ashkon Shaahinfar, Sarah E Kellner, Nayana Dhavan, Timothy P Williams

**Affiliations:** 1 Department of Global Health and Population, Harvard School of Public Health, 651 Huntington Avenue, Boston, MA 02115, USA; 2 Research Program on Children and Global Adversity, François-Xavier Bagnoud Center for Health and Human Rights, Harvard University, 651 Huntington Avenue, Boston, MA 02115, USA; 3 Department of Social and Policy Sciences, University of Bath, Bath, UK

**Keywords:** India, Labor migration, Child protection, Case study, SAFE model

## Abstract

**Background:**

Many of India’s estimated 40 million migrant workers in the construction industry migrate with their children. Though India is undergoing rapid economic growth, numerous child protection issues remain. Migrant workers and their children face serious threats to their health, safety, and well-being. We examined risk and protective factors influencing the basic rights and protections of children and families living and working at a construction site outside Delhi.

**Methods:**

Using case study methods and a rights-based model of child protection, the SAFE model, we triangulated data from in-depth interviews with stakeholders on and near the site (including employees, middlemen, and managers); 14 participants, interviews with child protection and corporate policy experts in greater Delhi (8 participants), and focus group discussions (FGD) with workers (4 FGDs, 25 members) and their children (2 FGDs, 9 members).

**Results:**

Analyses illuminated complex and interrelated stressors characterizing the health and well-being of migrant workers and their children in urban settings. These included limited access to healthcare, few educational opportunities, piecemeal wages, and unsafe or unsanitary living and working conditions. Analyses also identified both protective and potentially dangerous survival strategies, such as child labor, undertaken by migrant families in the face of these challenges.

**Conclusions:**

By exploring the risks faced by migrant workers and their children in the urban construction industry in India, we illustrate the alarming implications for their health, safety, livelihoods, and development. Our findings, illuminated through the SAFE model, call attention to the need for enhanced systems of corporate and government accountability as well as the implementation of holistic child-focused and child-friendly policies and programs in order to ensure the rights and protection of this hyper-mobile, and often invisible, population.

## Background

In India, there are an estimated 40 million migrant laborers in the construction industry [1], who together make an immense contribution to the country’s rapidly developing economy [2]. Many of these laborers are parents who migrate with their young children to work and live in very challenging conditions [3-5]. Despite a broad epidemiological “migration and health” literature [6] and the documented impact of labor migration on child health [7,8], including explorations of child maltreatment in migrant families [9-12], little research has examined the health, safety, development, and well-being of migrant workers’ children. Using the methodology of a social science case study [13] conducted at a large construction site in the National Capital Region near Delhi, India, we examine dynamics influencing the security and well-being of migrant children and families who live near and work in infrastructure development projects.

### Public health implications of migration for children and families

Migrants worldwide comprise a heterogeneous population that includes more than 214 million international and 740 million internal migrants [14]. The reasons for migration are diverse: for some, it is necessitated by civil conflict, natural disaster, development, or trafficking; while for others, it is out of desperation to escape profound poverty. The field of public health has traditionally focused on how mobile populations contribute to communicable disease epidemiology e.g. [15]. There is, however, a growing literature on migration and health, which addresses a variety of topics including: mental health [16], reproductive health [17], maternal and child health [18,19], tobacco and substance use [20,21], occupational health [22], and child abuse and neglect e.g. [9]. Studies have generally indicated that the drivers of migration, such as socioeconomic status [7,8,23-25], closely determine migrant health, rather than the process of migration itself. Increasingly, research has highlighted structural and institutional factors that affect migrant health, such as denial of medical care and its relationship to child survival [7].

Migration has implications for family well-being, including the safety, development, and education of children of migrant workers. Parental absence and struggle for survival have been tied to harmful socio-emotional impacts on children left behind [26-28]. With regards to the education of children of migrant families, researchers have found both positive impacts from remittances [29,30] and negative effects due to lack of parental support and supervision [26,31] as well as disincentivization by the prospect of migration [32]. Researchers seeking to understand the complex connections between family migration and child abuse have also highlighted poverty and socioeconomic stress [9,10,33,34], as well as social isolation and lack of social capital [11,12,33-35]. Frequent mobility has also been correlated with child maltreatment within families [36,37] as well as in neighborhoods and communities [38-40].

### Labor migration and construction in India

The drivers and consequences of labor migration in India are as diverse as its regions and peoples [41]. While rural–urban and interstate migration make up relatively small portions of all migration in India (18% and 13% of 315 million migrants, respectively, per the 2001 census) [42], rapid development has increased these numbers. By recent trade union estimates, there are approximately 40 million interstate migrants in the construction industry alone [1]. This group of internal migrant workers, defined in the Interstate Migrant Workmen Act of India as “any person who is recruited by or through a contractor [including middlemen] in one State under an agreement or other arrangement for employment in an establishment in another State”, and their families, comprise the general focus of the present study [43].

Cyclical migration has long been an important livelihood strategy for the rural poor of India [44,45]. Seasonal climate fluctuations in regions such as the flood-prone Ganges basin and the rain-dependent semi-arid tropics make for agrarian lifestyles fraught with risk and food insecurity [4,46,47]. Landlessness and social-deprivation [3,48,49], indebtedness [4,50], and limited employment opportunities [44,49,50] all drive individuals and families to migrate.

The impact of migration on families and communities varies. While longitudinal field studies in India indicate improved wages and income among migrants over time [2,48,51], the most economically and socially-deprived have remained in debt [50,52]. Deshingkar and colleagues summarized their observations in Madhya Pradesh: “…for the poorest groups of migrants, especially unskilled and uneducated Scheduled Castes (SCs) and Scheduled Tribes (STs) who still migrate through agents, or who cannot enter remunerative … work because of discrimination, working conditions and earnings are far from ideal and positive changes in living standards are less certain and slower” [2]. Though some studies suggest migrants are more able to resist exploitation [2,51], recent reports document illegally low wages and strenuous work hours [4,53,54].

An emerging body of literature documents numerous threats to the health and well-being of migrant children in India, including poor living conditions [3-5,54], frequent injuries of workers [1,4], poor access to drinking water [4,5,53], and sexual violence towards women and children [53,55]. Furthermore, urban migrant laborers have great difficulty accessing government programs otherwise accessible in rural settings, including those for health care and insurance [4,54,55], childcare [4,54,56], education [53,56], and food rations [5,53,54]. However, the interrelated and interdependent relationship of these threats to child health and well-being is often overlooked.

### Conceptual framework

A rights-based, holistic model of child security, the SAFE model, provided the conceptual basis for the present study. Situating child protection within the nested social ecologies of families, communities, and the larger political, cultural and historical context, the SAFE model examines interrelatedness among four core domains of children’s basic needs and rights: **S**afety/freedom from harm; **A**ccess to basic physiological needs and healthcare; **F**amily and connection to others; **E**ducation and economic security [57]. Of central importance to SAFE is the idea that insecurity in any of these fundamental domains threatens security in the others. The SAFE model posits that in the face of child security threats, children and families demonstrate considerable agency, adopting survival strategies to meet their basic security needs. These survival strategies may take risky forms (with cascading negative effects on other dimensions of child security and well-being) or adaptive forms [57]. For instance, to overcome family economic insecurity, some families may give their child over to bonded child labor while others may organize workers’ collectives to secure a loan to start a small business. The purpose of the SAFE model is to identify and build on adaptive strategies while also highlighting risky strategies in order to enact preventive interventions, provide alternatives, or end third party manipulation [58].

Fundamental concepts of the United Nations Convention on the Rights of the Child (CRC) [59], such as the evolving capacities of the child and the interdependence and interrelatedness of child rights and basic security needs, are implicit to the SAFE model. The SAFE domains also map onto rights delineated in the CRC, such as the rights to life, survival, and development (e.g., Art. 6); education (Art. 28); health (Art. 24); family connections (e.g. Arts. 9, 20); and protection from violence (Art. 19) as well as various other special protection articles (e.g. Arts. 32–40) [59].

## Methods

### Case study design and research questions

We applied a case study methodology [13] to identify factors affecting children’s security and well-being at a construction site in the National Capital Region of India using the SAFE model as a theoretical framework for our data collection. A case study approach has particular utility in addressing the “how” and “why” of contemporary phenomena within their real life contexts [13]. Furthermore, this approach allowed for an in depth examination of issues related to child protection on a single site and the resulting elucidation of complex phenomena. As with prior applications of the SAFE model e.g. [58], we specifically sought to illuminate child protection threats facing children in families migrating for work in India’s construction industry with particular attention to the SAFE domains and their interrelatedness. We also sought to identify adaptive and dangerous survival strategies used by migrant families. The study was guided by four research questions: (1) What conditions lead children and families to the site?; (2) What are the security threats facing children at the site as described by children, adults, local community representatives, and child protection stakeholders in the National Capital Region?; (3) How do children and families of the site cope with or respond to security threats and situations of adversity?; (4a) In what ways, if any, do public and private sectors of civil society work to support the security and well-being of migrant children and families on the site?; and (4b) More generally, how do local stakeholders think public and private sectors can promote the security and well-being of migrant children and families, if at all?

Our study methods placed particular emphasis on triangulation of information pertaining to the study site, and included key informant interviews, focus groups discussions, ethnographic observations, and a thorough exploration of relevant peer-reviewed and grey literatures. The research was facilitated by Mobile Crèches, a non-governmental organization (NGO) in Delhi that works with the developer at the construction site to provide an early childhood development-oriented crèche and daycare center to care for migrant children during the work day. Indian law requires the establishment of a crèche at sites with more than 50 women [60], but the law is rarely implemented [61] (Table 1).

**Table 1:** **Site demographics**^***a***^

**Number of families**^***b***^		**46**
Number of children < 12^*b*^	Age (years)	
	*0–3*	28
	*3–6*	27
	*6–12*	23
	*Total*	78
Number of children attending Mobile Crèches center^*b*^	*0–3*	27
	*3–6*	26
	*6–12*	23
	*Total*	76
Average duration of attendance at Mobile Crèches center (child-days per child)^*c*^		48

^*a*^Data courtesy of Mobile Crèches.

^*b*^Sept. – Oct. 2010.

^*c*^April – Sept. 2010.

### Study sample and recruitment

The research team worked closely with Mobile Crèches to select and recruit study participants. Purposive sampling was used to select participants from the construction company leadership, government, civil society, as well as migrant workers and children living and working at the site. We conducted six focus groups, including: two with female workers (N = 15, median age 26), one with male workers (N = 5, median age 35), one with adult “Malda workers” (described below; N = 5, median age 25), one with young boys (N = 4, median age 11), and one with young girls (N = 5, median age 7). A small group of children and adolescents, who were on the site without their parents, were deemed by the research team in discussion with the host NGO too vulnerable for involvement and were excluded from the study. Each focus group consisted of four to ten people and lasted from one to two hours. We also conducted seven in-depth interviews with providers who work with the children at and near the site (e.g. school teachers and child care providers; see Table 2). Six developer and contractor representatives at various levels of leadership and employment as well as a local business owner were also interviewed. In order to locate this case study within a larger political context, we also spoke with eight key informants from the government and international and local NGOs.

**Table 2:** Sources of data

7 KI interviews in corporate sector	construction supervisor, crèche supervisor, 2 general managers (1 on-site, 1 from separate development company), Malda *jamadar*, adult Malda worker, local business owner
2 KI interviews with local schools	principal at government school for girls, teacher at private school
5 KI interviews with crèche staff and local NGO	crèche staff including: supervisor, center in charge, doctor, part-time employee/construction worker; coordinator of local NGO
6 Focus group discussions	male construction workers (N = 5); adult Malda workers (N = 5); female construction workers (N = 10); female construction workers (N = 5); male children of migrant workers (N = 4); girl children of migrant workers (N = 5)
Observations	construction site, communal living area, surrounding community
8 KI Interviews with child protection stakeholders in the National Capital Region	5 interviews with local and international NGOs, 2 interviews with UN agencies, 1 interview with government official
38 participants of validity-strengthening “member checks” (e.g. follow-up meetings, focus group discussions, and interviews - both new and previous respondents)	female construction workers (N = 17), male construction workers (N = 13, including 1 *jamadar*), male and female children (N = 4), and previous key informants in the National Capital Region (N = 4)

### Data collection

Primary data were collected in the months of January and July 2010, with additional “member checks” (described below) in January-February 2011. All interviews and focus groups were conducted in Hindi or English by local research assistants trained in research ethics and qualitative interviewing techniques. Working in pairs, one research assistant conducted the interview while the other took detailed field notes. All focus groups were held in the local language (Hindi), and key informant interviews were held in English or Hindi. Focus groups and interview guides were open-ended in nature (e.g., “What problems, if any, do children and families face in this site?”), and interviewers probed to gain further insight into emerging issues. Depending on respondent preference, interviews were recorded digitally or via detailed note-writing. All interview and focus group data were transcribed, de-identified, and stored electronically with access limited to authorized research staff, ensuring participant confidentiality. Local research assistants worked in small teams to make accurate Hindi to English translations. Ethical approval was obtained from both the Human Subjects Committee of the Harvard School of Public Health and a local Community Advisory Board in Delhi. Verbal informed consent and independent child assent was obtained from all participants. Parental consent for their children’s participation was obtained at least one day prior to the child focus groups. Participants were given opportunities to ask the local research team any questions before, during, and after the focus group discussions.

### Data analysis

Our method of qualitative data analysis included open coding, category construction, and axial coding to examine relationships between categories consistent with a grounded theory-based analysis [62] and Thematic Content Analysis (TCA) [63]. This approach entailed a 4-stage procedure: 1) We first conducted an open-coding process of all data using both SAFE model informed categories as well as findings arising organically from the data. 2) Categories and themes that were most saturated in the data informed the development of a coding scheme, e.g., “poverty”, “access to medical care” “hunger”. 3) To examine reliability, two team members trained in the coding scheme independently coded 10% of transcripts. The code book was refined and reliability testing was repeated until all coding was at 80% reliability. 4) Using the code book, the qualitative dataset was coded in Nvivo 8 [64], a qualitative data analysis program.

After multiple readings of the data, emergent themes were identified and specific codes representing core themes or phenomena were developed. The codebook underwent several iterations with input from multiple coders, allowing its structure to be refined and adapted over time [65]. This approach was further supplemented by axial coding [62] to examine the interrelatedness between key concepts and identify cross-cutting themes. In January-February 2011, members of the research team returned to Delhi to re-contact several study participants and to undertake follow-up interviews and focus group discussions through a validation exercise known as “member checks” [66] (N = 38 participants).

## Results

### Factors driving family migration to construction site

The laborers, many from the rural areas of distant states such as Bihar and West Bengal (see Table 3), likened their experience as a migrant worker to “going abroad” or living in *pardes* (foreign land). “*If a person has … everything, then why would they come here in this jungle to live*”, explained one female laborer (age 45). “*[Only] [t]he ones who have some problem or are suffering would come here*”. Most laborers and families came to the construction site through *jamadars* or *thekedars* from their village, middlemen hired by contractors to sort out logistics related to recruitment, transport, and, in some cases, on-site accommodation. Others arrived through previously migrated family members or through direct company recruitment. A number of respondents had recently migrated for the first or second time: “*It’s been five days since I arrived here. I have just come from home. Earlier when I was here, I stayed for nine months*”. (female laborer, age 21). Many had spent years in *pardes*, moving from work site to work site, often in association with the same company. As one male worker described, “*We came here and have stayed here since 1995. And we used to go to our native place and come back. I go once or twice in a year to my village. Again, I come to join the same construction firm*” (age 55). Citing the costs and risks associated with travel, many migrants would visit the village infrequently. One group of male laborers referred to as “Malda workers” work on short-term, 50-day contracts, which permit them to pay off small debts, work in their agricultural “off season”, and return home (typically Malda and Cooch Behar districts of West Bengal) to their families and land regularly. No indication of forced labor, coercion, or human trafficking emerged from our observations or discussions with children and adults.

**Table 3:** **Source of construction labor migration to National Capital Region, India (2010–2011)**^***a***^

*Madhya Pradesh*	31%
*Bihar*	22%
*Chhattisgarh*	19%
*West Bengal*	13%
*Odisha*	7%
*Uttar Pradesh*	6%
*Jharkhand*	3%
*Rajasthan*	1%

^*a*^Data courtesy of Mobile Crèches, from sample of 2804 families from construction sites with Mobile Crèches centers in the National Capital Region, India; April 2010 – March 2011.

Frequently cited drivers for migration included the opportunity to “earn and eat”, family indebtedness, limited land ownership, and poor fertility of land in villages. Laborers shared stories of migration to meet basic needs for their families: “*Earlier I was a tailor master in Malda. I owned a shop … but I left the work as my youngest daughter was sick. I have spent so much money on her treatment … I was running out of money*” (male supervising laborer). One mother explained, “*We have three children two boys and one girl. We have a house that is made up of mud that is falling down. With the thought to re-build our house and to educate my children we have come here*” (age 25). Another described how her family was compelled to migrate, “*I do not have fields, and there are no rains. In the village, we were dying from hunger and thirst, so we have left children there and have come only with one son to work here on this site*”. (age 30). Still, others cited their intent to help secure a better life for their children. A female migrant (age 22) explained, “*We are poor. We are taking care of, raising our children … to make them move forward, we are earning money. If we educate our children, we will make them into something*”. All of the women in our focus groups were parents; two-thirds of these mothers had children living with them and their husbands on the site, nearly half of whom also had children living with grandparents in the village.

### Safety and freedom from harm at the construction site

Displayed prominently at the entrance of the construction site was a sign that read, “*Parents must warn children that this is an unsafe area*”. Large machinery, moving vehicles, and precariously-situated construction materials were ever-present on the site. Children, in particular, expressed worry about their parents’ safety. They recalled incidents that had occurred at other sites where children or workers had been severely hurt or killed, resulting in a preoccupation with the safety of their parents: “*When people work in this site … [they] climb from a rope … then they fall, then people die”*, said a girl, age 8. In fact, between July 2010 and January 2011, two workers had died in falls on the worksite.

### Access to basic physiological needs: housing, food insecurity and accessing medical care

#### Living conditions & basic amenities

Workers and their families lived in cramped, temporary structures, termed *jhuggis,* made of either corrugated tin or brick and mortar. During the course of our fieldwork, entire portions of the housing area were destroyed and moved due to expanding construction on the site. Many families spent their day off rebuilding or tending to their shelters, a time-consuming and labor-intensive task. While the site manager cited the danger electricity would pose in the tin *jhuggis*, the lack of electricity for fans or other cooling systems proved especially burdensome and potentially dangerous given high humidity and temperatures exceeding 100°F in the summer months: “*Poor people come from far off and feel so hot here. We stay inside and are drenched in sweat, but still they do not provide us with any electricity…*” (female worker, age 35). To cope with the extreme heat, particularly when the crèche was closed, children would spend time in half-built, multi-story towers where, as one boy (age 12) put it, “*winds … [give] people a calm and cool situation”*. Unsupervised, they were placed at risk of falling from the towers or being injured.

The quality and availability of basic amenities, including housing, access to drinking water, affordable food, and proper sanitation were also of primary concern to participants. Many children expressed nostalgia for their village life, citing the heat, cramped *jhuggis*, and lack of open space. One girl (age 7) insisted, “*Everyone, boys and girls, says it is better back home*”. Although workers were generally satisfied with the availability of clean water, some reported conflicts over communal pipelines. Food was also readily available near the site; however, given their lack of access to ration cards and the high price of food around Delhi, some migrants struggled to afford food and needed to purchase it from the grocery shop owner, or *lala,* on credit. Sanitation at the site was observed to be poor, with latrines that were often full and seldom cleaned. The lack of separate facilities for men and women was distressing for female workers. Despite these various concerns, the multiple layers of accountability and relative powerlessness of workers meant change was unlikely; as one man (age 55) lamented, “*We cannot do anything. If we will ask, we will be kicked out of this place*”.

#### Health & healthcare

Many migrate to a worksite in hopes of improving family well-being; yet the health challenges they encounter in pursuing this survival strategy may put children and families at further peril. Respondents familiar with the site described health issues ranging from water-borne and other infectious diseases to heat-exhaustion, dehydration and other work-related problems. Unresolved malnutrition and anemia were both common among children [67]. While the crèche offered some medical support for children and their families, there were divergent opinions about whether the company or contractors provided care for workers’ children. One male worker (age 35) asserted that there was “*[n]othing for the children*” and that “*the facilities are only for the workers*”. Conversely, a supervisor for a sub-contractor claimed that their facilities were available to both workers and their families. Options for workers themselves were also limited as employers only took responsibility for provision of, transportation to, and payment for care related to work place injury or illness. Basic first aid was provided on the construction site and in the crèche, and an ambulance was reportedly available around the clock. However, in case of off-duty health problems among workers or of more serious injuries or illnesses among children, workers and families had to seek off-site care and pay out of pocket. Informed choice of providers was uncommon, and many participants reported going to informal practitioners of herbal and other alternative medicine as well as unlicensed providers without any medical background in the nearby market. One female worker (age 25) lamented, “*Here there are doctors, some are good and some are bad … there is no fixed doctor, so what do we do? [When we’re] in trouble, we have to go back to our village*”. Primary care services, such as adult health screening, mental health services, and chronic disease management, did not appear readily available.

The costs of paying for healthcare, medications, and other associated expenses (e.g. transportation) were a resounding concern among workers. As one woman (age 35) explained, “*If [the doctor] gives us two injections, it costs us Rs.200 [$4.45 USD]. We are already in so much debt, and if we are not well, how would we go to work?*” Missed work meant lost wages for ill workers, posing additional risk to their families. To save time, many visited private hospitals rather than seek the largely free services at a government hospital, which was reportedly farther away. By law, the contractors are responsible for covering costs of work-related injuries; however, as a manager with the development company admitted, *“…not everyone gets to take advantage of the contractor all risk policy because migrants are … a floating population and are not necessarily recognized by the contractor*”. Similarly, due to barriers to registering as local labor welfare board beneficiaries or obtaining documentation of Below Poverty Line (BPL) status, very few workers had access to locally-implemented health insurance schemes intended to provide financial assistance for hospitalizations and chronic care. Thus, in the face of high healthcare costs, workers often turned to their *jamadars* or *thekadars* for loans and accompaniment to medical care, adding potentially substantial debt which could force them to extend their work or contract periods.

### Family and connection to others: limited monitoring and constant pressure

Construction work meant that many parents were spending long hours without direct capacity to monitor their young children at the site, which became additionally challenging without the presence of extended family. The existence of a functioning crèche at the site greatly mitigated some of the risks due to parental inability to monitor their children. According to both male and female laborers, sexual violence was of diminishing concern. This may be linked to a trend towards leaving school age girls with extended family in the village. One crèche staff member reflected on this survival strategy: “*I saw that earlier young girls [migrated] with their parents, but many crimes occurred like assault … When they went back to their village, they talked about these problems, and awareness increased. Now they leave their young girls at their home”*. Issues of family conflict arose infrequently in focus groups and interviews. However, the use of alcohol, and cramped living spaces, were both mentioned as linked to domestic and intimate partner violence. A local teacher, who noted that children were at times reluctant to go home due to violence, reflected on these factors: “*Society demands certain things from kids, and parents have zero economic power. Sometimes they beat kids and women when children ask for their needs to be met”.* Child labor was also a seldom-raised topic, but in addition to the adolescent Malda workers and a few teenage children working on the site with their parents, a number of children worked at the worksite canteen, businesses in the nearby market, and in rag-picking in exchange for food and shelter. These children were some of the most vulnerable in this site. We were unable to interview these children and adolescents without being observed by their supervisors and were unable to obtain parental consent given limited information about their family members.

### Education and economic security: looking to the future

Children at the site faced numerous barriers to education and learning due to issues of distance, expense, lack of documentation, and frequent movement. A few older boys and one girl were able to attend a nearby private school due to reduced-payment options that were offered for children of migrant workers. However, the nearest government school was far away and across a busy highway. While a few children did attend, responses suggested it was too dangerous to access for many children at this site, thus impeding their right to universal primary education [68]. Speaking to the de facto discrimination faced by migrant children without residency documentation, the manager of a development company explained, “Although the schools don’t have official rules against [migrant] children, they are not willing to admit children who do not have some degree of permanency”. Even when a child was able to enroll in school, a wide range of obstacles hindered learning, including: the lack of electricity and thus lighting in the *jhuggis,* a generally poor study environment at home, inability to afford or access tutors, as well as domestic responsibilities that led to school absenteeism. As a local teacher at a private school observed, “*Our kids who are migrants, they really struggle. They have a really difficult time because they compare themselves to [non-migrant] kids*”. These prevailing concerns around education were tied to most parents’ decision to leave their school-aged children (particularly girls) with extended family in the village, allowing uninterrupted schooling. As one man (age 35) asserted, “*We cannot keep our children here; otherwise their education will spoil*”. Not surprisingly, most school-age children on the site were not in school. Apparently having no other safe place to go, many spent their days in the crèche.

#### Work & wages

Financial concerns were at the forefront of discussion for adult migrant workers. Despite the site manager’s assurance that workers “*do not express concern over wages*”, construction workers told a story of working extended hours for low, piecemeal wages and having only every other Sunday off. Malda workers instead received payment in advance of their 50-day contract period and had no days off. Many workers claimed that payments for piecemeal work and other wages could be delayed, even for months, although not universally reported. However, workers did commonly describe an improvement in wages and financial circumstances relative to opportunities in the village. Nonetheless, many workers, particularly unskilled laborers, were still struggling to provide for their families, much less to save earnings. As one mother (age 35) explained, “*We have to take care of the whole family; we came here to save money. If he would increase the rate, we would save a little. The amount we earn goes to eating*”. In response to these difficulties, delayed payments, and other unexpected costs, respondents reported borrowing money from family, friends, coworkers, or their *jamadars* or *thekadars*. Parents also worked overtime to provide for and meet the basic needs of their families. In contrast to other sites, where participants described that women are generally subject to reprimands and deductions in wages for breastfeeding, this site was notably sensitive to the needs of breastfeeding mothers, and women did not report difficulty feeding their infants. Yet, given that the law allows only two nursing breaks per day The Maternity Benefits Act, [69], section 11, crèche employees needed to provide supplemental formula feeding. Meanwhile, a number of children in the crèche were being monitored for mild and moderate malnutrition and were provided with nutritional supplements.

### Protective processes

A sense of community solidarity, social support, and the presence of the crèche were all important sources of risk mitigation and protection at the site. Despite difficulties with wages, living conditions, health care, and access to other services, conflicts among workers did not appear to be common or significant. A crèche employee observed that workers “… *become friends and … live like brothers and sisters*”. With many workers living together in “lanes” based on their language and region of origin, communal living also appeared to transcend some of the tension that could otherwise arise from differences in origin, caste, and religion. As one female worker (age 33) explained, “*We are all laborers and live together with each other happily. Every one helps each other*”. Although relationships among migrants were often brief, workers were able to rely upon their community support by turning to one another in times of need. A few respondents suggested their personal sense of faith by invoking God’s role in protecting their children or allowing their family’s success, representing an emergent and preliminary theme.

The crèche also served a protective role that had broader implications for families. One of its primary functions was to provide a safe and caring environment for children during the day, allowing both mothers and fathers to work. As one mother (age 22) noted, “*[Without] such a service or facility, where would we have left our children? Children would come after us to the site … How would we earn our livelihood then?*” The crèche provided basic health care services and regular doctor visits (including growth-monitoring, immunizations, and de-worming), meals to children during the day, and informational sessions for parents on issues like school enrollment. Although not designed to replace school, it also provided younger children an opportunity to develop cognitive, motor, and language skills as well as classroom discipline important for school readiness. The crèche also served to free older siblings from the task of childrearing. The crèche was a safe, child-friendly haven in an otherwise harsh physical environment, in utter contrast to most construction sites, where, as one key informant described, “*the young child is invariably either left alone, unattended, or in the care of siblings … [and] the implications for a child’s development can easily be gauged”*. The NGO also worked to mainstream older children into local schools and to encourage provision of basic amenities at this site.

### Invisible children, invisible families: the key informant perspective

Our key informants from government agencies and non-governmental organizations reflected upon a scenario in which vulnerable and hyper-mobile migrant families are surrounded by the complex layers of accountability of a rapidly expanding Indian economy. Grappling with the confluence of challenges facing migrant workers and their children, our respondents emphasized the obligations of local and national governments as well as corporations towards the fulfillment of the rights of this marginalized and often “invisible” population.

As described in the Indian labor migration literature, our key informants discussed the overlapping factors that both drive already vulnerable families towards migrant labor and make them more vulnerable in the process. One labor expert highlighted the role of discrimination and lack of economic opportunity: “*I would describe this as compounding vulnerabilities … Indeed, the caste system is there; there are other minority groups that are discriminated against too. For example, some Muslim communities – many of them are what we call “backwards” [per the “Other Backward Classes” classification]. There are absolutely no opportunities for these people; they have no hope for the future and are therefore quite vulnerable”.* Another respondent from a child protection NGO saw migration as leveling the playing field of vulnerability across castes: “*Once they come here, the vulnerabilities are quite the same for them as migrants … Except that of economic status – may be able to afford services … Looking at similar economic standard with people migrating, the vulnerabilities are very similar*”.

Hypermobility poses particular challenges to migrant children’s education. As local NGO stakeholder explained: “*We have very bright children who study in the village but who have migrated or have shifted schools four or five times a year, and they are in an inappropriate class because they have missed so much. And there is no scope for them to ever get on with the education and ever do anything but manual work in the future*”. Furthermore, though Indian policy prohibits requiring children to present birth or transfer certificates in order to access a new school Right of Children to Free and Compulsory Education Act, [68], lack of awareness and compliance poses an illegal barrier to migrant children’s right to education. A child protection expert explained: “*A school teacher unaware that this is a law, would continue to insist on these certificates. So that would be the work of a civil society in informing the school teacher that you can now not stop a child on any excuse”.* Amidst so many obstacles, according to our respondents, migrant families further face corporations and governments unwilling to take accountability for fulfilling their rights. One construction site general manager discussed the lack of incentive in this area: *“There is competition between different contractors for jobs; the bidder with the lowest price generally wins the contract. As companies reduce their budgetary requirements to stay competitive, one of the first things to go is often the amenities provided for children and workers*”. One key informant explained that destination states often see migrants as a drain on local resources and thus hesitate to take responsibility for them. As a development expert elucidated, “*It’s possible that within the next two or three years, the government may think seriously about working on some strategies to reach them. But as of now they are invisible. They are neither in the rural planning, neither in the urban planning—nobody talks about them. They are in a limbo”*. Such planning is further complicated by the difficulty of precisely capturing complex and varied migration patterns in the census.

In order to improve accountability and uphold existing policy, local experts offered various suggestions, including: maximizing interstate coordination; developing memoranda of understanding among development stakeholders with a monitoring agency; granting corporate and government contracts to builders only upon provision of required services (e.g. crèche); and empowering and educating workers and children to advocate for their rights. As one key informant advised, “*If migration must happen … then we must take steps to make it safer. There should be outreach to communities of origin [and] dialog and information-sharing with subcontractors, with children and families, about their legal right for safety and fair treatment*”. Another respondent argued against use of the term “corporate social responsibility”, remarking, “*If they would just follow the law, that would be fine*”.

## Discussion

Migrant work is a survival strategy that poses many threats to healthy childrearing. Utilizing the SAFE model as a lens towards child protection, this study yielded in-depth insight into the complex dynamics affecting the security and well-being of migrant children and families at a construction site near Delhi. Families encountered ongoing economic insecurity and a host of other risks for child protection, including: children living and playing in unsafe areas and potentially facing violence at home (**S**afety); limited access to appropriate medical care as well as risks of malnutrition (**A**ccess to basic physiological needs); reduced parental monitoring, family separation, and trade-offs between time with children and the need to work (**F**amily connection); and limited school access for school-age children as well as a tenuous economic state (**E**ducation/Economic Security). These basic security domains were also interrelated and interdependent. In general, as explained by Orellana and colleagues, migrating “families who are pressed for household survival do not have the luxury to foreground children’s ‘developmental needs’” [28], p. 587. For instance, the family’s decision to migrate to secure their economic situation often had consequences for their children’s access to school, which increased an intergenerational cycle of “distress migration”. Similarly, a mother’s need to work and the limited options for exclusive breastfeeding likely heightened children’s risk of diarrheal illness and respiratory infections [70-72] as well as risk of malnutrition [73]. Furthermore, vulnerable families migrating to “earn and eat” and meet their basic needs, encountered an unforgiving urban landscape that posed new and unique risks to children’s security and affronts to their basic rights. As Rogaly and Rafique have described in the context of seasonal migration in West Bengal, many of the families in our study had no hope to save or pay off debt and were simply in “a struggle to stand still” [52].

This case study has exemplified a clear need to work expediently towards the realization of the rights of this hyper-mobile, invisible population. To this end, it is necessary to both fill gaps in Indian legislation and to improve the implementation and enforcement of existing laws. A recent report by the National Commission for Enterprises in the Unorganized Sector, noted that the vulnerability of migrant workers and their children stems largely from “the lack of or ineffective implementation of … [as well as] … lack of awareness among the workers regarding existence of the laws” meant to ensure their entitlements and protect their well-being [56], p. 165. Mechanisms for ensuring accountability and implementation are not apparent in the law governing corporate responsibility in India, especially in the unorganized sector.

One crucial gap in government planning and implementation relates to migrant rights to identity and documentation. For migrant workers in the unorganized sector, these rights have particular salience to the ability to access legal entitlements. In Indian policy, this is delineated as contractors’ responsibility to issue passbooks to migrant workers in the Inter-state Migrant Workmen Act [IMWA, 43, article 12] as well as the directive of state labor welfare boards to maintain registrations and provide identity cards for workers (who have worked 90 days) in the Building and Other Construction Workers Act [BOCWA, 60, articles 12–13]. Despite these laws and efforts like India’s Unique Identification Card pilot project, obtaining such documentation is often a challenge due to migrants’ transitory status and short-term residency. This lack of documentation impedes access to various basic services at migrants’ destination, including food rations and children’s education. While India’s Right of Children to Free and Compulsory Education Act [68] dictates that children should at no time be turned away for lack of documents, study findings suggest the clause lags in implementation. Disruptions to children's educational experience may have implications for their lives and future livelihoods.

While responsibility for providing services and protections is delineated in law and policy, lack of enforcement leaves many corporations shirking responsibility for providing for their workers and their families. For example, the BOCWA describes responsibilities for construction project employers (e.g. government-owned entities, private developers and sub-contractors), to provide drinking water, latrines and urinals, accommodations, first aid, canteens, and other services to mothers and their families. With accountability diffused so broadly across different stakeholders at the study construction site, viewpoints expressed across various layers of management were at best disconnected from and at worst disingenuously ignorant of the daily realities of migrant workers and their families. However, while there is also the requirement for provision of childcare for children under six at sites with more than 50 women, this task is not explicitly assigned to the employer nor is it enforced. In the site where the present case study was situated, the crèche played a fundamental role in nurturing child development and monitoring child safety and nutrition. Sector-wide provision of such services, implemented by corporate actors in partnership with local governments, would fill a critical void in the rights of migrant children, with long-term benefits to their health and capability [74-78].

Clearly, the government plays a crucial role in delineating the obligations of corporations, ensuring these obligations are met, and shaping policy that affects migrant families. Using the IMWA and BOCWA as well as myriad other broader labor policies as guidelines, each state’s labor welfare board has flexibility to implement their own rules and local schemes. For instance, the local labor welfare board near our case study site recently announced, though has yet to implement, a school transportation scheme for children of construction workers. Similarly, large sums of funds collected from corporations according to the Building and Other Construction Workers’ Welfare Cess Act [79] have yet to be used for worker welfare schemes.

Moreover, the Government of India has developed cross-sectoral efforts and government-civil society partnerships to protect children from maltreatment and exploitation, such as the newly implemented Integrated Child Protection Scheme (ICPS). With explicit mention of children of migrant families, this policy aims to “create a protective environment by improving regulatory frameworks, strengthening structures and professional capacities at national, state, and district levels so as to cover all child protection issues and provide child friendly services at all levels” [80]. The ICPS includes provisions to counter some of the barriers to protecting marginalized or vulnerable children and should include those living and even working on construction sites. In the years ahead, implementation of the ICPS in India has the potential to reduce risks to child health and development by addressing vulnerabilities across the SAFE domains examined here.

### Limitations

This study intended to capture an in-depth understanding of the complex dynamics facing migrant families in one construction site in India. While the analysis was successful in this regard, several limitations should be acknowledged. First, the use of a case study methodology limits our ability to generalize to other construction sites locally or nationally in India. While the external validity of the study is limited in this manner, the internal validity of our qualitative approach was bolstered by the inclusion of a range of stakeholders as well as the triangulation of key informant interviews, focus groups, observations, and document review. Along this same theme, our study focused on the status of children and families living and working on the construction site with minimal probing regarding the children left at home in the village. These children may face yet uncovered and significant health and protection risks; more research focused on these populations is required. Second, the existence of a crèche at the site studied allowed us to gain access to the site and our participants, but may also have successfully mitigated the intensity of some of the child protection threats that might be present in sites without a crèche. We would hypothesize that a site managed by a developer receptive to providing a crèche, such as the one used in this study, might generally be more child-, family-, and worker-friendly than those sites that do not have a crèche. However, despite an estimated 40 million migrant laborers in India’s construction industry alone, the law requiring crèches at construction sites is rarely implemented, and most sites would likely paint a much worse picture. Finally, though various techniques in data collection and analysis were utilized to minimize biased responses (such as monitoring dominant respondents, assuring confidentiality, triangulating with on-site observations, and conducting member checks), the sensitive nature of focus group discussions and the relative powerlessness of migrant workers may have limited the sensitivity for uncovering the full depth of adversity faced by families on the site. Nonetheless, our discussions with workers and their children yielded a substantial variety of child protection issues and related coping mechanisms, which did not vary significantly by respondent.

## Conclusions

As the Indian economy continues to expand and urbanize, labor migration will undoubtedly continue as a mode of subsistence or survival among rural families. Through analyses using the SAFE model of children's security, this study provides a lens for viewing the constraints faced by migrant children and families who undertake a broader array of survival strategies to cope with their circumstances. It demonstrates the feasibility and utility of taking this holistic and human rights-based approach to child protection analyses. In light of the complex conditions facing this hyper-mobile population, findings suggest policymakers, corporations, and civil society must work to develop initiatives to implement and enforce the rights of migrant workers and their children to identity, family, health, safety, development, education and economic security. The Government of India is certainly legally bound by the Convention on the Rights of the Child to protect these rights for all children, without discrimination based on gender, caste, or other status [59]. Corporations must similarly be compelled to ensure the well-being of India’s future human capital by promoting child-friendly development.

## Competing interests

The authors declare that they have no competing interests.

## Authors’ contributions

TSB conceived of the study and participated in its design and coordination along with TW and AS. TW and AS conducted the primary qualitative fieldwork. TSB, ND, and AS conducted the validity-enhancing member checks. All authors, including SK, contributed to the qualitative data analysis and helped draft and finalize the manuscript. All authors read and approved the final manuscript.

## Pre-publication history

The pre-publication history for this paper can be accessed here:

http://www.biomedcentral.com/1471-2458/13/858/prepub
